# Microstructural Degradation of the AlMo_0.5_NbTa_0.5_TiZr Refractory Metal High-Entropy Superalloy at Elevated Temperatures

**DOI:** 10.3390/e23010080

**Published:** 2021-01-08

**Authors:** Tamsin E. Whitfield, Howard J. Stone, C. Neil Jones, Nicholas G. Jones

**Affiliations:** 1 Department of Materials Science and Metallurgy, University of Cambridge, Cambridge CB3 0FS, UK; tw398@cam.ac.uk (T.E.W.); hjs1002@cam.ac.uk (H.J.S.); 2 Rolls-Royce plc, Derby DE24 8BJ, UK; colin.jones@rolls-royce.com

**Keywords:** refractory metal high entropy alloys, phase transformation/precipitation, intermetallics, thermodynamics

## Abstract

Refractory metal high-entropy superalloys (RSA), which possess a nanoscale microstructure of B2 and bcc phases, have been developed to offer high temperature capabilities beyond conventional Ni-based alloys. Despite showing a number of excellent attributes, to date there has been little consideration of their microstructural stability, which is an essential feature of any material employed in high temperature service. Here, the stability of the exemplar RSA AlMo_0.5_NbTa_0.5_TiZr is studied following 1000 h exposures at 1200, 1000 and 800 °C. Crucially, the initial nanoscale cuboidal B2 + bcc microstructure was found to be unstable following the thermal exposures. Extensive intragranular precipitation of a hexagonal Al-Zr-rich intermetallic occurred at all temperatures and, where present, the bcc and B2 phases had coarsened and changed morphology. This microstructural evolution will concomitantly change both the mechanical and environmental properties and is likely to be detrimental to the in-service performance of the alloy.

## 1. Introduction

The aerospace and power-generation industries are striving to meet demanding targets for more efficient, lower emission gas turbine engines [1,2,3,4]. To achieve greater efficiency with current engine architectures it is necessary to raise the turbine entry temperature [5]. Modification of the operating conditions in this way presents a significant challenge to the components in the turbine section of these engines and places an even more demanding set of requirements on the underlying materials. Crucially, the current state-of-the-art materials, Ni-based superalloys, are limited by their comparatively low melting points and, consequently, are unlikely to be able to support the desired increase in operational temperatures [6,7]. Therefore, new material options are needed that offer high temperature mechanical and environmental properties capable of superseding Ni-based superalloys.

However, peak operating temperature is not the only factor that needs to be considered when evaluating a material for elevated temperature service. For a typical flight cycle, the time spent at maximum thrust is relatively short, with far longer durations spent in the less demanding cruise condition. As such, the vast majority of an aeroengine turbine component’s service life is spent at intermediate temperatures. As a result, in addition to being able to withstand short bursts at peak operating temperatures, it is critical that the performance of a high temperature alloy does not degrade during extended exposures at the lower temperature experienced during cruise. Since an alloy’s properties are intrinsically linked to its microstructural condition, maintaining performance under these operating conditions requires a high level of microstructural stability. Therefore, characterising the microstructural stability of an alloy both at peak and average service temperatures is crucial to evaluating its suitability for high temperature service.

The recent development of refractory metal high-entropy superalloys (RSA), based on the AlMoNbTaTiZr system, has attracted significant attention as they have the potential for use in structural applications at temperatures higher than currently accessible [8,9]. Initial results have shown that these RSA have certain advantages over current Ni-based superalloys, including lower densities and higher compressive strengths [7]. In the homogenised condition, the microstructures of RSA typically exhibit a nanoscale two-phase basketweave structure. This fine scale structure consists of refractory metal-rich particles, which have a bcc crystal structure, within an ordered B2 superlattice matrix phase. In addition, the presence of an Al-Zr-rich intermetallic phase is widely reported, with this phase delineating the grain boundaries of the matrix phase [7,10,11,12].

This two-phase intragranular microstructure is often considered to be analogous to the γ and γ′ microstructures of Ni-based superalloys [13]. However, there are some important differences that need to be highlighted. First, whilst both types of material contain two phases that are crystallographically related, the actual crystal systems are different. In Ni-based superalloys, the γ matrix phase has a disordered fcc crystal structure whilst the γ′ precipitates that form within it have the ordered L1_2_ superlattice structure. As outlined above, the phases present in RSA are based on the bcc system and, therefore, fundamental differences in behaviour will exist between the two types of material as a result of the different atomic coordination. Second, as outlined in previous statements, the arrangement of the phases within the microstructures is also different. The matrix phase of the Ni-based superalloy is the more ductile disordered phase, whilst in RSA the matrix is the ordered phase. As such, despite the attractive high temperature strengths, the ordered matrix phase and the presence of Al-Zr intermetallics leads to very limited room temperature ductility and the implications that this has for toughness is a key concern for potential applications [10]. Significant efforts have already been made to invert the microstructure of RSA and suppress the formation of the Al-Zr-rich grain boundary phase, details of these studies can be found elsewhere [10,14,15,16].

Despite its critical importance to potential applications, little is known about the thermal stability of RSA microstructures. Relatively few studies have considered thermal exposures at temperatures below the B2 solvus and, even then, the reported durations are relatively short [7,10,11,13,14,16,17,18,19]. It is well known from Ni-based superalloys that microstructural changes, such as coarsening of the reinforcing features or the precipitation of additional intermetallic phases, can occur during long duration thermal exposures, to the detriment of the environmental and mechanical properties. As such, evaluating the susceptibility of the finescale RSA microstructures to similar degradation is another key aspect in assessing their suitability for high temperature service.

Recently, we reported on the microstructural evolution of two RSAs, AlMo_0.5_NbTa_0.5_TiZr_0.5_ and AlNbTa_0.5_TiZr_0.5_, following 1000 h exposures at 1200, 1000 and 800 °C [20]. The initial microstructures, which comprised homogeneous B2 grains delineated by an Al-Zr-rich grain boundary intermetallic phase, evolved during the exposures. Significant coarsening of the Al-Zr phase on the grain boundaries occurred, along with precipitation of the same phase within the B2 grains. Furthermore, a number of additional phases also formed in AlNbTa_0.5_TiZr_0.5_, which would further alter in-service performance [20]. However, rather surprisingly, there are very few data relating to the microstructural stability of the archetypal B2 + bcc RSA, AlMo_0.5_NbTa_0.5_TiZr. To date, the most pertinent information comprises the microstructural condition following a 100 h heat treatment at 1400 °C, where the Al-Zr-rich intermetallic phase coarsened [12], and following isothermal deformation at 1000 °C, where the finescale bcc + B2 structure was retained throughout processing [7]. Despite these observations, neither study provides suitable data to make an assessment of the microstructural stability of the alloy, particularly as no information relating to intermediate temperatures is available. As such, to address this need, here, we report on the microstructural evolution of AlMo_0.5_NbTa_0.5_TiZr following 1000 h exposures at 1200, 1000 and 800 °C.

## 2. Materials and Methods

A 40 g ingot of AlMo_0.5_NbTa_0.5_TiZr (20Al–10Mo–20Nb–10Ta–20Ti–20Zr at.%) was fabricated via arc melting of elemental metals (≥99.9% purity) under an inert Ar atmosphere that had been pre-gettered by melting a piece of pure Ti. To enhance the macroscopic homogeneity, the RSA ingot was inverted and re-melted five times. The material was subsequently homogenised at 1400 °C for 24 h in a zirconia crucible encapsulated within a quartz tube, which had been evacuated and backfilled with 99.999% purity Ar. Following homogenisation, the sample was cooled at 10 °C/min to 750 °C and then furnace cooled to room temperature. Long duration thermal exposures of 1000 h were conducted on ~10 × 7 × 7 mm sections of the homogenised material at temperatures of 1200, 1000 and 800 °C. For all exposures, the samples were wrapped in a Ta foil getter and sealed in quartz tubes under vacuum. At the end of the exposure duration, samples were quenched into ice water within their quartz tubes to avoid oxidation.

Microstructural characterisation was performed using scanning electron microscopy (SEM) using specimens prepared through standard metallographic techniques, which culminated in polishing using a buffered 0.06 µm colloidal silica solution. Back-scattered electron (BSE) images and energy-dispersive X-ray (EDX) elemental distribution maps were acquired using a Zeiss GeminiSEM 300 equipped with an Oxford Instruments X-Max^N^ 50 detector. To assess phase fractions, large area BSE images were thresholded and quantitatively analysed using the ImageJ software. X-ray diffraction (XRD) patterns were obtained using a Bruker D8 diffractometer in Bragg-Brentano configuration, with Ni filtered Cu radiation, between angles of 10 and 130° 2θ. These data were fitted using a Pawley refinement in TOPAS Academic. Thermodynamic modelling of the phase equilibria, as a series of equilibrium calculations across the experimentally studied temperature range was conducted in ThermoCalc using the SSOL5, TCTI2, TCHEA1, TTTI3 and PanTi databases.

## 3. Results and Discussion

In the as-cast state, the material comprised refractory metal-rich dendrites surrounded by an Al-Zr enriched interdendritic constituent. To remove this micro-segregation, the material was homogenised at 1400 °C for 24 h, in line with previous studies [7,11,18]. Following homogenisation, EDX elemental distribution maps showed an even contrast over the length scale of the prior dendrites, indicating that the solidification-induced micro-segregation had been eliminated. Quantitative EDX analysis showed the alloy composition to be 18.4Al–8.8Mo–19.3Nb–10.5Ta–21.0Ti–21.9Zr at.%, consistent with the intended composition.

**Figure 1:**
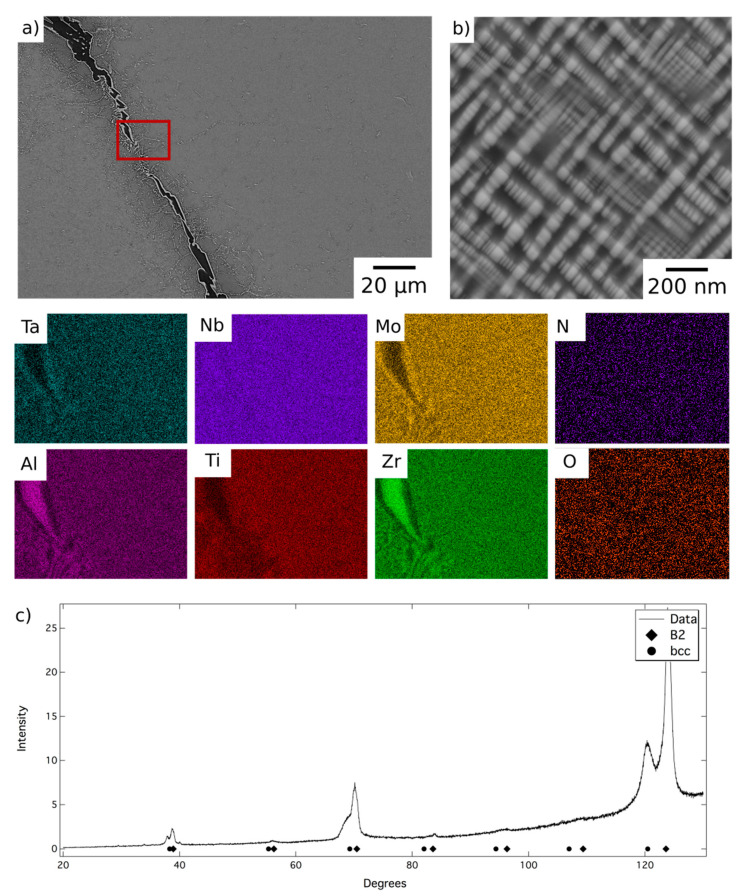
Back-scattered electron (BSE) micrographs of AlMo_0.5_NbTa_0.5_TiZr following homogenisation heat treatment (**a**) at lower and (**b**) at higher magnification, and EDX elemental distribution maps corresponding to the region of the BSE image indicated with a red box. (**c**) The corresponding X-ray diffraction pattern acquired from material in this condition.

BSE micrographs of the alloy following homogenisation heat treatment are shown in Figure 1. At low magnification, Figure 1a, the microstructure comprised relatively large grains, with a hatched contrast indicating finer internal structures, delineated by a dark BSE contrast phase along the grain boundaries. The EDX elemental distribution maps indicated that the dark contrast phase was enriched in Al and Zr and quantitative compositional analysis is presented in Table 1. The composition, morphology and location of these features are very similar to those reported in previous work and, therefore, it is believed that this is the Al_4_Zr_5_ hexagonal intermetallic (P6_3_/mcm, a = 8.31 Å, c = 5.52 Å) identified in references [12,20]. A layer of a bright contrast phase can be seen along the surface of the dark contrast phase, which did not occur in the previous reports of this alloy, but similar features were observed in AlMo_0.5_NbTa_0.5_TiZr_0.5_ at temperatures where the refractory metal rich phase was present [20]. Given the slow furnace cool experienced by AlMo_0.5_NbTa_0.5_TiZr following homogenisation, this bright contrast layer is likely to have formed on the surface of the Al-Zr rich phase during cooling.

At higher magnification, a two-phase nanoscale microstructure was observed within the grains, as shown in Figure 1b. This microstructure comprised bright BSE contrast cuboidal precipitates in a darker contrast matrix, in keeping with previous reports of AlMo_0.5_NbTa_0.5_TiZr [7,10,11,12]. XRD data acquired from material in this condition, shown in Figure 1c, contained reflections from two sets of closely related cubic structures with lattice parameters of 3.27 Å and 3.32 Å. These lattice parameters were in very good agreement with those identified previously for AlMo_0.5_NbTa_0.5_TiZr, suggesting the presence of the bcc and B2 phases reported elsewhere [7,10,12,13]. Additional peaks corresponding to the Al-Zr intermetallic were not present with sufficient intensity to enable characterisation of this phase. This is unsurprising given that this phase resided along the grain boundaries and hence, given the large grain size present, did not amount to a significant area fraction of the sample. However, small additional reflections were present in the diffraction data, for example at 40° 2θ, which were consistent with the positions expected from previous characterisation of this phase [12]. Therefore, despite having been produced through a different route, it is believed that the microstructural condition of the homogenised material, which is the baseline for the current work, is the same B2 + bcc microstructure identified in previous studies [12,13].

**Figure 2:**
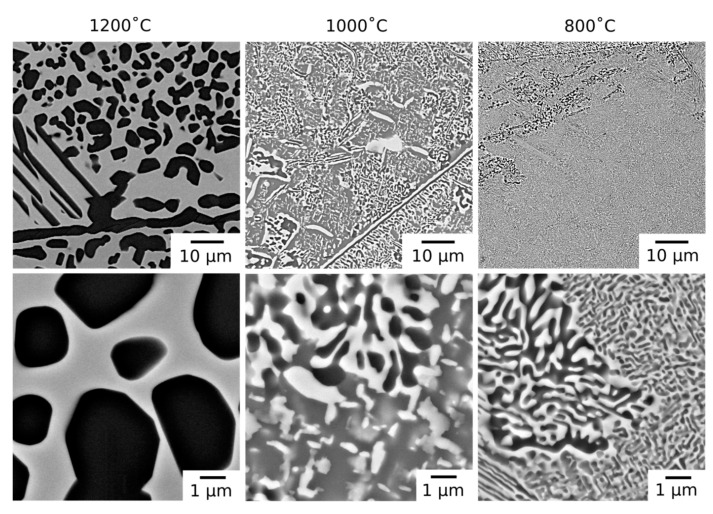
BSE micrographs of AlMo_0.5_NbTa_0.5_TiZr following thermal exposure at 1200, 1000 and 800 °C for 1000 h at lower magnification (**top**) and higher magnification (**bottom**).

Following long duration exposure, the microstructure of the material was observed to have evolved at all of the temperatures investigated. An overview of the resulting microstructures can be seen in Figure 2. In all of the samples, the dark BSE contrast phase remained present along the grain boundaries. In addition, new intragranular precipitates with similar dark BSE contrast were observed in both rounded and elongated morphologies. Notably, the microstructures from exposure at 1000 °C and 800 °C, which were extremely similar, also contained a significant volume fraction of bright contrast intragranular precipitates.

**Figure 3:**
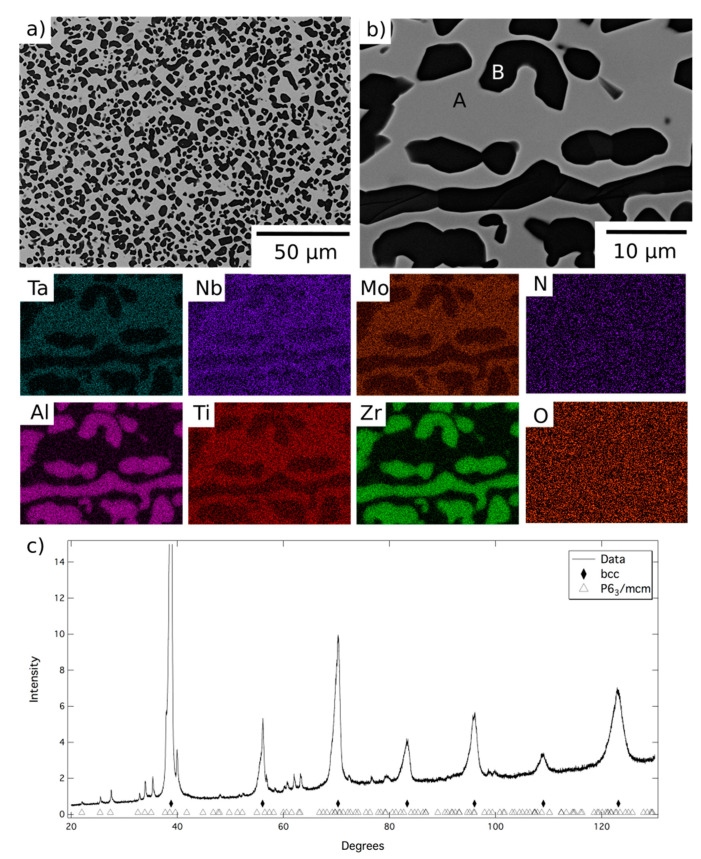
BSE images (**a**) at lower and (**b**) at higher magnification of AlMo_0.5_NbTa_0.5_TiZr following exposure at 1200 °C for 1000 h and EDX elemental distribution maps corresponding to the top right BSE image. (**c**) The corresponding X-ray diffraction pattern acquired from material in this condition.

The microstructure resulting from exposure at 1200 °C can be seen in more detail in Figure 3, along with corresponding EDX elemental distribution maps and XRD data. BSE images indicated the material comprised a grey contrast matrix (labelled A), within which relatively coarse angular particles with a dark contrast (labelled B) had formed. Quantitative image analysis indicated that the volume fraction of these precipitates was significant, approaching 50%. As mentioned above, the dark contrast phase observed at the grain boundaries of the homogenised material was retained following the 1200 °C exposure. However, no evidence of a nanoscale two-phase microstructure, similar to that shown in Figure 1b, was found within the matrix regions of this sample.

The EDX elemental distribution maps showed Phase A to be enriched in Mo, Nb, Ta and Ti, whilst the dark contrast phase, both on the grain boundaries and within the grain interiors, was Al-Zr-rich. Quantitative assessment revealed that the composition of the grain boundary and intragranular precipitates were very similar and, therefore, that just two phases were present at 1200 °C (compositional data listed in Table 1). The XRD data, Figure 3c, indicated the presence of a bcc phase and a P6_3_/mcm phase, with refined lattice parameters of a = 8.06 and c = 5.44 Å. Given the compositional and structural similarities, it is believed that Phase B is the same hexagonal P6_3_/mcm Al-Zr intermetallic reported in similar RSA studies [10,12,20].

Following exposure at 1200 °C, Phase A was significantly enriched in Mo, Nb and Ta, whilst simultaneously depleted in Al and Zr, when compared to the intergranular regions of the homogenised material. These changes are consistent with the precipitation of a large volume fraction of the Al-Zr intermetallic. However, such compositional changes will also influence the structure of Phase A and the likelihood of forming the nanoscale two-phase microstructure shown in Figure 1b. The enrichment in refractory metal elements and reduction in Al is likely to stabilise a bcc structure [21]. In addition, since the formation of the nanoscale microstructure is primarily driven by the miscibility gap between Zr and refractory metals, the very low Zr content of the matrix phase is unlikely to result in such a phase separation [10,22,23]. These assertions are supported by other reports of the phase chemistries and structures identified in other RSA. The composition of Phase A lies within the range commonly reported for bcc phases and has significantly lower Al and Zr than recorded in the B2 phases in references [12,15,20]. Furthermore, including the B2 phase in the XRD analysis did not improve the refinement significantly. The absence of the B2 phase in these samples implies that 1200 °C is above the solvus temperature, removing its contribution to the mechanical properties of the alloy at this temperature. As such, it is concluded that at 1200 °C the material is in a two-phase field bounded by the bcc and Al-Zr based intermetallic phases.

The microstructures obtained following 1000 h exposures at temperatures ≤1000 °C contained both bright and dark BSE contrast precipitates within the grain interior, Figure 2. In some regions, the precipitates formed interpenetrating networks with lamellar morphologies. The size of these regions and the lamellae within them were largest near the grain boundaries and, where coarse, the dark contrast phase appeared to be encased in a layer of the bright contrast phase, a morphology reported in other RSAs [20,21]. Away from these regions, the bright precipitates had a rounded, rod-like morphology. This contrast to the bright cuboidal particles observed in the homogenized material may be attributed to the increased length scale of the precipitates following exposure at 1000 °C, which reduces the effect of surface energy and, hence, the effect of crystallographic symmetry on the precipitate morphology. Such rod-like morphologies in bcc/B2 systems have been attributed to the agglomeration of nanoscale cuboidal particles, where sufficient diffusion times allows for coarsening [24].

**Figure 4:**
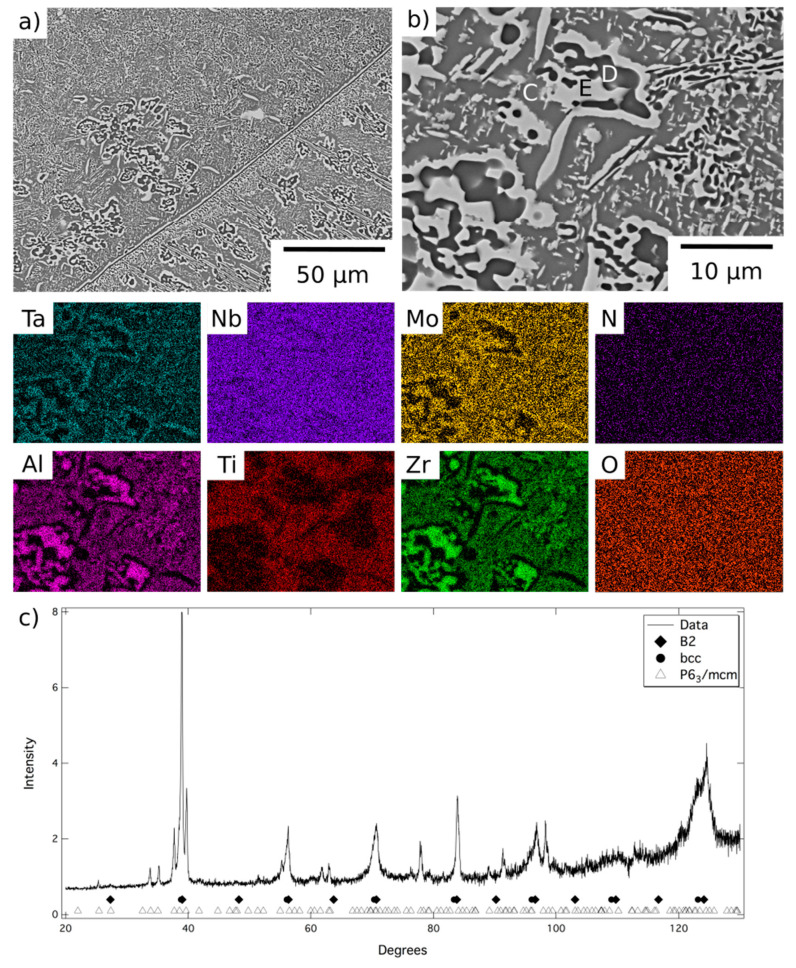
BSE images (**a**) at lower and (**b**) at higher magnification of AlMo_0.5_NbTa_0.5_TiZr following exposure at 1000 °C for 1000 h and EDX elemental distribution maps corresponding to the top right BSE image. (**c**) The corresponding X-ray diffraction pattern acquired from material in this condition.

**Figure 5:**
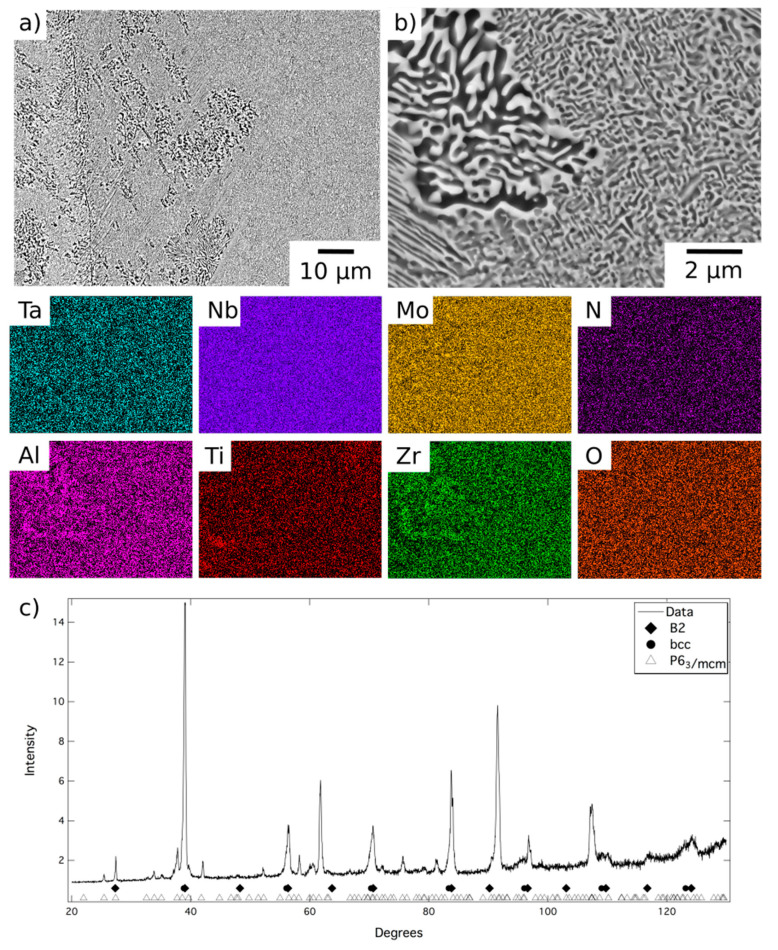
BSE images (**a**) at lower and (**b**) at higher magnification of AlMo_0.5_NbTa_0.5_TiZr following exposure at 800 °C for 1000 h and EDX elemental distribution maps corresponding to the top right BSE image. (**c**) The corresponding X-ray diffraction pattern acquired from material in this condition.

A representative area from the material exposed for 1000 h at 1000 °C is shown in Figure 4, along with corresponding EDX elemental distribution maps and XRD data. These data were very similar to those obtained from the material exposed for 1000 h at 800 °C, Figure 5, but, due to the fine scale of the microstructure, it was not possible to acquire distinct elemental partitioning data for the phases present, as it was beyond the resolution of the instrument. The grey contrast matrix (labelled C in Figure 4) exhibited a multi-element composition, with no unique strong preferential partitioning. The dark contrast precipitates (labelled D) were once again observed to be Al-Zr-rich and depleted in Mo, Ta and, to a lesser extent Nb. The bright contrast phase (labelled E) exhibited the opposite partitioning trends, being enriched in refractory metal elements and depleted in Al and Zr. Quantitative analysis of these data is provided in Table 1. The XRD data, Figure 4c and Figure 5c, contained reflections corresponding to bcc, B2 and P6_3_/mcm structures, the latter of which had refined lattice parameters of a = 8.09, c = 5.47Å after exposure at 1000 °C and a = 8.10, c = 5.46 Å after exposure at 800 °C.

Comparing the results from the different exposures it is evident that the refractory metal-rich features, Phase A and Phase E, are the same bcc structured phase, whilst the Al-Zr-rich features, Phases B and D, are the same hexagonal intermetallic phase. Therefore, Phase C, which was slightly enriched in Al, Ti, and Zr and only observed following the lower temperature exposures, must correspond to the B2 structure. These observations are in line with reports from similar RSA within the literature [10,12,15,20] and indicate that at temperatures ≤1000 °C the alloy was in a three-phase field. At 1200 °C, only the bcc and hexagonal intermetallic phases were present and, therefore, heat treatments above this temperature reside in a two-phase, bcc + hexagonal intermetallic phase field. Consequently, the precursor to the nanoscale B2 + bcc microstructure, which forms on quenching from homogenisation at temperatures >1200 °C, is confirmed to have a bcc structure with the B2 phase forming through a spinodal decomposition plus ordering mechanism [15,21].

Understanding the effect of composition on the microstructures and phase equilibria of RSA is paramount for the development of future alloys. Therefore, it is notable that the alloy studied in this work is closely related to AlMo_0.5_NbTa_0.5_TiZr_0.5,_ which has also been studied following long duration exposures [20]. Crucially, the increased Zr content of AlMo_0.5_NbTa_0.5_TiZr, studied here, resulted in several key differences. A greater volume fraction of the Al-Zr-rich intermetallic formed in AlMo_0.5_NbTa_0.5_TiZr, as is seen most clearly at 1200 °C where the volume fraction was approximately double that observed in AlMo_0.5_NbTa_0.5_TiZr_0.5_. Consequently, at 1200 °C the matrix phase in AlMo_0.5_NbTa_0.5_TiZr was significantly depleted in Al and enriched in Mo and Ta when compared to that in AlMo_0.5_NbTa_0.5_TiZr_0.5_. Hence, it is believed that following cooling to room temperature the composition of the matrix phase in AlMo_0.5_NbTa_0.5_TiZr meant that it maintained a bcc structure, unlike the matrix phase of AlMo_0.5_NbTa_0.5_TiZr_0.5,_ which was shown to have a B2 structure following cooling from the exposure temperature. The formation of the B2 phase also indicates that sufficient Al concentration must have been retained within the matrix of AlMo_0.5_NbTa_0.5_TiZr_0.5_ to facilitate ordering [6,8,9,21,23,25]. The final, important, difference between these two studies was present within the microstructures following exposure at 800 °C. Despite AlMo_0.5_NbTa_0.5_TiZr_0.5_ not forming a nanoscale cuboidal B2 + bcc microstructure on cooling from homogenisation, nanoscale cuboidal bcc precipitates were observed following exposure at 800 °C and, therefore, are believed to be stable at these temperatures. Whereas AlMo_0.5_NbTa_0.5_TiZr, which exhibited a nanoscale B2 + bcc microstructure following cooling from homogenisation, had a microstructure dominated by the Al-Zr intermetallic at 800 °C and did not show the B2 + bcc cuboidal nanostructures. Consequently, high Zr contents may be disadvantageous for RSA design as they favour the formation of the Al-Zr-rich intermetallic phase.

**Figure 6:**
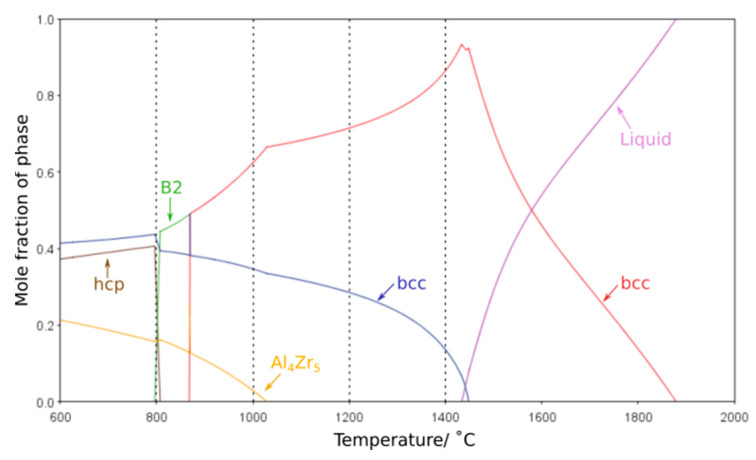
ThermoCalc equilibrium phase volume fraction predictions of AlMo_0.5_NbTa_0.5_TiZr over a temperature range of 600–2000 °C created using SSol5 database. The heat treatment temperatures examined experimentally are indicated by vertical dashed lines.

One of the most critical features for any high-temperature material is the stability of its microstructure whilst in service. Any significant evolution in the size or morphology of the constituent features or the formation of additional phases will lead to an associated change in properties. Thus, it is of great importance that the phase equilibria at elevated temperatures can be accurately predicted by thermodynamic calculations. Modelling of the phase equilibria across the temperature range studied with several different databases suggested that the high temperature phases were not maintained at lower temperatures. However, it should be noted that each database predicted markedly different phase equilibria, all of which exhibited significant deviations from the experimental findings. In particular, the Al-Zr intermetallic phases predicted varied, along with the temperature ranges over which they were indicated to be stable. Furthermore, the B2 phase was not predicted by many of the databases and, when present, was typically only projected to form at temperatures of 900 °C and below, in contrast to the results herein, where the B2 phase is seen following exposure at 1000 °C. This is consistent with reported difficulties in predicting phases within refractory metal high entropy systems, and critically predicting the B2 phase, on account of the limited data and issues in extrapolating from the bounding binary systems as discussed in references [6,9,26,27,28,29] The most closely matching result was achieved with the ThermoCalc SSol5 database, which demonstrated reasonable agreement to the phases observed, albeit with large differences in solvus temperatures, most notably for the Al_4_Zr_5_ intermetallic, Figure 6. The discrepancies between thermodynamic calculations and experimental observations highlight that further database development is required before such outputs can be considered truly predictive for RSA systems and aid future alloy design. However, refinement of the thermodynamic databases requires high-quality experimental data corresponding to the equilibrium state of these alloys at a range of different temperatures. As such, it is vital that the community continues to perform long duration studies, with detailed microstructural characterisation, so as to provide the results that will enable thermodynamic predictions with greater fidelity.

## 4. Conclusions

The principal observation from the long duration exposures performed here is the instability of the nanoscale cuboidal B2 + bcc microstructure when AlMo_0.5_NbTa_0.5_TiZr was exposed at temperatures between 800 and 1200 °C. Of particular concern is the extensive intragranular formation of the hexagonal Al-Zr based intermetallic, which is likely to be detrimental to the mechanical performance of the alloy [10,30,31] and potentially compromise other properties through elemental redistribution [32]. As such, based on the present results, it seems unlikely that this particular alloy would be suitable for high temperature service in a gas turbine engine, although the impact of the observed microstructural evolution on the alloy’s performance needs to be fully evaluated.

The observed phase equilibria indicate that at 1200 °C the alloy is in a two-phase field comprising a bcc solid solution and the Al-Zr intermetallic phases. This observation is significant as it demonstrates that the B2 phase is not stable to very high temperatures and must form during cooling from a bcc precursor.

Looking forwards, it is likely that new alloy compositions will be required and given the key role of the Al-Zr-based intermetallic phase in the current microstructural evolution, future RSA chemistries may need to be carefully balanced to avoid or minimise its formation. In this work, the raised Zr content when compared to AlMo_0.5_NbTa_0.5_TiZr_0.5_ was found to favour the formation of a greater volume fraction of this phase. Furthermore, thermodynamic predictions for these systems do not currently offer high fidelity. Therefore, it is critical that further experimental studies of phase equilibria are performed, not only to assess the microstructural stability of a given alloy but also to provide the underlying data that will enable the refinement of the current thermodynamic databases.

## Figures and Tables

**Table 1:** Quantitative energy-dispersive X-ray (EDX) data obtained from AlMo_0.5_NbTa_0.5_TiZr following homogenisation heat treatment at 1400 °C and subsequent thermal exposures at 1200 and 1000 °C for 1000 h. All concentrations in at%, with the uncertainties being the standard deviations in the measurements.

Phase	Al	Mo	Nb	Ta	Ti	Zr	Structure
**Homogenised**							
**Grain interior**	17.2 ± 0.4	8.9 ± 0.3	19.3 ± 0.3	10.1 ± 0.2	22.0 ± 0.1	22.5 ± 0.5	B2 + bcc
**Grain boundary**	30.4 ± 1.0	3.1 ± 0.5	11.4 ± 0.6	4.0 ± 0.4	11.0 ± 0.4	40.1 ± 1.3	P6_3_/mcm
**1200 °C**							
**Phase A**	7.5 ± 0.1	16.3 ± 0.9	28.0 ± 0.4	17.9 ± 0.0	26.8 ± 0.4	3.6 ±0.3	bcc
**Phase B**	33.8 ± 0.8	1.4 ± 0.5	9.9 ± 0.7	3.2 ± 0.5	12.6 ± 0.8	39.1 ± 0.7	P6_3_/mcm
**1000 °C**							
**Phase C**	20.6 ± 0.1	6.4 ± 0.1	18.0 ± 0.9	7.0 ± 0.3	24.3 ± 0.9	23.7 ± 0.3	B2
**Phase D**	30.8 ± 1.0	2.6 ± 0.6	11.6 ± 0.2	4.8 ± 0.4	12.7 ± 0.5	37.5 ± 1.0	P6_3_/mcm
**Phase E**	8.6 ± 1.6	16.0 ± 0.6	27.6 ± 1.0	18.0 ± 1.3	20.9 ± 0.8	8.9 ± 1.5	bcc

## Data Availability

The underlying research data can be accessed from The University of Cambridge repository using the following link: https://doi.org/10.17863/CAM.62758.
